# Supplementary material for: Characterization, Stress Response and Functional Analyses of Giant River Prawn (Macrobrachium rosenbergii) Glucose-Regulated Protein 78 (Mr-grp78) under Temperature Stress and during Aeromonas hydrophila Infection
Source: Animals (Basel). 2021 Oct 19;11(10):3004. doi: 10.3390/ani11103004 (PMC8532774; doi:10.3390/ani11103004)
Supplement: Supplementary file 1 [file animals-11-03004-s001.zip › animals-1332940-supplementary.pptx]

## Slide 1
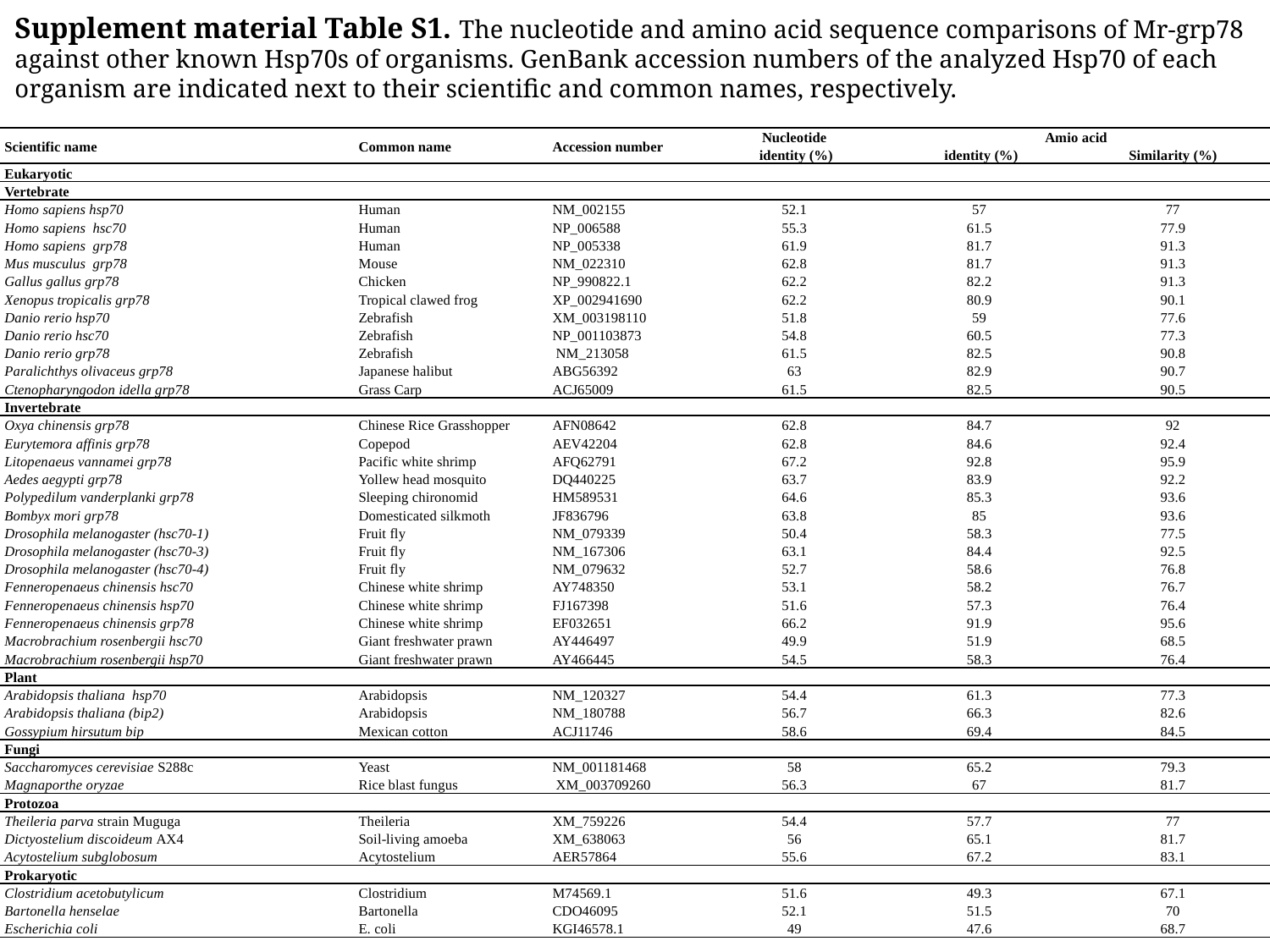

Supplement material Table S1. The nucleotide and amino acid sequence comparisons of Mr-grp78 against other known Hsp70s of organisms. GenBank accession numbers of the analyzed Hsp70 of each organism are indicated next to their scientific and common names, respectively.
| Scientific name | Common name | Accession number | Nucleotide | Amio acid | |
| --- | --- | --- | --- | --- | --- |
| | | | identity (%) | identity (%) | Similarity (%) |
| Eukaryotic | | | | | |
| Vertebrate | | | | | |
| Homo sapiens hsp70 | Human | NM\_002155 | 52.1 | 57 | 77 |
| Homo sapiens hsc70 | Human | NP\_006588 | 55.3 | 61.5 | 77.9 |
| Homo sapiens grp78 | Human | NP\_005338 | 61.9 | 81.7 | 91.3 |
| Mus musculus grp78 | Mouse | NM\_022310 | 62.8 | 81.7 | 91.3 |
| Gallus gallus grp78 | Chicken | NP\_990822.1 | 62.2 | 82.2 | 91.3 |
| Xenopus tropicalis grp78 | Tropical clawed frog | XP\_002941690 | 62.2 | 80.9 | 90.1 |
| Danio rerio hsp70 | Zebrafish | XM\_003198110 | 51.8 | 59 | 77.6 |
| Danio rerio hsc70 | Zebrafish | NP\_001103873 | 54.8 | 60.5 | 77.3 |
| Danio rerio grp78 | Zebrafish | NM\_213058 | 61.5 | 82.5 | 90.8 |
| Paralichthys olivaceus grp78 | Japanese halibut | ABG56392 | 63 | 82.9 | 90.7 |
| Ctenopharyngodon idella grp78 | Grass Carp | ACJ65009 | 61.5 | 82.5 | 90.5 |
| Invertebrate | | | | | |
| Oxya chinensis grp78 | Chinese Rice Grasshopper | AFN08642 | 62.8 | 84.7 | 92 |
| Eurytemora affinis grp78 | Copepod | AEV42204 | 62.8 | 84.6 | 92.4 |
| Litopenaeus vannamei grp78 | Pacific white shrimp | AFQ62791 | 67.2 | 92.8 | 95.9 |
| Aedes aegypti grp78 | Yollew head mosquito | DQ440225 | 63.7 | 83.9 | 92.2 |
| Polypedilum vanderplanki grp78 | Sleeping chironomid | HM589531 | 64.6 | 85.3 | 93.6 |
| Bombyx mori grp78 | Domesticated silkmoth | JF836796 | 63.8 | 85 | 93.6 |
| Drosophila melanogaster (hsc70-1) | Fruit fly | NM\_079339 | 50.4 | 58.3 | 77.5 |
| Drosophila melanogaster (hsc70-3) | Fruit fly | NM\_167306 | 63.1 | 84.4 | 92.5 |
| Drosophila melanogaster (hsc70-4) | Fruit fly | NM\_079632 | 52.7 | 58.6 | 76.8 |
| Fenneropenaeus chinensis hsc70 | Chinese white shrimp | AY748350 | 53.1 | 58.2 | 76.7 |
| Fenneropenaeus chinensis hsp70 | Chinese white shrimp | FJ167398 | 51.6 | 57.3 | 76.4 |
| Fenneropenaeus chinensis grp78 | Chinese white shrimp | EF032651 | 66.2 | 91.9 | 95.6 |
| Macrobrachium rosenbergii hsc70 | Giant freshwater prawn | AY446497 | 49.9 | 51.9 | 68.5 |
| Macrobrachium rosenbergii hsp70 | Giant freshwater prawn | AY466445 | 54.5 | 58.3 | 76.4 |
| Plant | | | | | |
| Arabidopsis thaliana hsp70 | Arabidopsis | NM\_120327 | 54.4 | 61.3 | 77.3 |
| Arabidopsis thaliana (bip2) | Arabidopsis | NM\_180788 | 56.7 | 66.3 | 82.6 |
| Gossypium hirsutum bip | Mexican cotton | ACJ11746 | 58.6 | 69.4 | 84.5 |
| Fungi | | | | | |
| Saccharomyces cerevisiae S288c | Yeast | NM\_001181468 | 58 | 65.2 | 79.3 |
| Magnaporthe oryzae | Rice blast fungus | XM\_003709260 | 56.3 | 67 | 81.7 |
| Protozoa | | | | | |
| Theileria parva strain Muguga | Theileria | XM\_759226 | 54.4 | 57.7 | 77 |
| Dictyostelium discoideum AX4 | Soil-living amoeba | XM\_638063 | 56 | 65.1 | 81.7 |
| Acytostelium subglobosum | Acytostelium | AER57864 | 55.6 | 67.2 | 83.1 |
| Prokaryotic | | | | | |
| Clostridium acetobutylicum | Clostridium | M74569.1 | 51.6 | 49.3 | 67.1 |
| Bartonella henselae | Bartonella | CDO46095 | 52.1 | 51.5 | 70 |
| Escherichia coli | E. coli | KGI46578.1 | 49 | 47.6 | 68.7 |
Supplement material Table S1 Srisapoome et al. (2021)
